# Adherence to Eye Drops Usage in Dry Eye Patients and Reasons for Non-Compliance: A Web-Based Survey

**DOI:** 10.3390/jcm11020367

**Published:** 2022-01-12

**Authors:** Miki Uchino, Norihiko Yokoi, Jun Shimazaki, Yuichi Hori, Kazuo Tsubota, on behalf of the Japan Dry Eye Society

**Affiliations:** 1 Department of Ophthalmology, Keio University School of Medicine, Tokyo 160-8582, Japan; tsubota@tsubota-lab.com; 2 Keishin Gotanda Eye Clinic, Tokyo 141-0022, Japan; 3 Department of Ophthalmology, Kyoto Prefectural University of Medicine, Kyoto 602-8566, Japan; nyokoi@koto.kpu-m.ac.jp; 4 Department of Ophthalmology, Tokyo Dental College Ichikawa General Hospital, Chiba 272-8513, Japan; meishano1@gmail.com; 5 Department of Ophthalmology, Toho University School of Medicine, Tokyo 143-8541, Japan; yhori@med.toho-u.ac.jp; 6 Tsubota Laboratory, Inc., Tokyo 160-0016, Japan

**Keywords:** dry eye disease, eye drop treatment, fixed use, subjective symptoms, web-based survey

## Abstract

This study aimed to investigate the actual use of eye drops for dry eye disease (DED), the reasons for instillation behavior, and the relationship between instillation behavior and subjective symptoms. This web-based cross-sectional study collected data on instillation behavior, medication instruction, reasons for instillation behavior, and subjective symptoms. In total, 2645 participants were enrolled. The proportion of participants who instilled at the frequency specified in the package insert (the specified frequency) was 10.2%. The most common reason for not instilling at the prescribed frequency was as-needed instillation to alleviate subjective symptoms, and 61.3% of participants instilled when feeling symptoms. The improvement in the subjective symptoms score was significantly greater in the group that regularly instilled at the specified frequency than the other group (*p* = 0.0027), and patients in the other group were younger and had a higher rate of contact lens use and over-the-counter eye drops use. In conclusion, most participants did not instill the DED eye drops at the specified frequency to alleviate symptoms. In order to obtain the appropriate effect of eye drops, ophthalmologists need to impress upon patients the importance of regular instillation at the frequency specified in the package insert, while taking into account patient characteristics.

## 1. Introduction

Dry eye disease (DED) is one of the most common clinically observed diseases [1]. The subjective symptoms of DED are various, and include irritation, dryness, eye fatigue, foreign body sensation, pain, heavy sensation, brightness, discharge, and fluctuating visual disturbances [2]. Additionally, DED symptoms remit and worsen repeatedly, and because it is difficult to cure DED, quality of life may decline [3].

DED is characterized by the vicious cycle of tear film instability and hyperosmolarity leading to ocular surface abnormality, which is thought to exacerbate disease severity [3,4]. Since many upstream risk factors are associated with unstable tear film layer, it is clinically difficult to remove all risk. Therefore, breaking the vicious cycle between abnormal functioning of the corneal epithelium and instability of the tear film layer and restoring the ocular surface and tear film integrity to the normal state may be important for improving DED symptoms.

The principal approach for treating DED is eye drops [5]. In Europe and the United States, anti-inflammatory therapies constitute the main treatments for DED; those approved for DED include cyclosporine (CsA) ophthalmic emulsion and lifitegrast ophthalmic solution 5.0% in North America, and CsA cationic emulsion in Europe [5]. Neither drug has been approved in Japan. Sodium hyaluronate ophthalmic solution (HA), 3% diquafosol sodium ophthalmic solution (DQS), and 2% rebamipide ophthalmic suspension (RBM) have been mainly used in Japan [6]. DQS stimulates the secretion of aqueous fluid and mucus directly on the ocular surface [7,8]. RBM increases the number of conjunctival goblet cells [9]. HA increases water retention in tears and accelerates corneal epithelium repair [10,11]. Therefore, these eye drops enable the stabilization of the tear film via targeted supplementation of deficient ocular surface components [12]. The main treatment method is anti-inflammatory therapy in Europe and the United States and tear film stabilization therapy in Japan. Though these treatments have different mechanisms of action, a single dose of these eye drops may temporarily relieve symptoms. However, when using eye drop treatments, it is necessary to regularly instill them at the frequency specified in the package insert (the specified frequency). On the other hand, DED is a disease with subjective symptoms [13], and instillation behavior may change in order to alleviate these symptoms. Depending on the subjective symptoms, the frequency of instillations per day may increase or decrease, and reaching the frequency of instillation required may not be possible. If the frequency of instillation is low, the effects of the drug will not persist and make it difficult to improve subjective symptoms. Given these considerations, it is very important to analyze the relationship between subjective symptoms and instillation behavior. However, the actual status of subjective symptoms and instillation behavior is not clear. Additionally, these instillation behaviors may be influenced by medication instructions, but no reports have examined the actual medication instruction and medication use situation in DED patients.

Therefore, in this large-scale, cross-sectional, web-based study, we investigated the actual frequency and patterns of DED eye drops usage for alleviating subjective symptoms, the reasons for non-compliant behavior in terms of medication instruction and subjective symptoms, as well as the characteristics of the patients and the improvement of subjective symptoms in the group that regularly instilled at the specified frequency (fixed-use group).

## 2. Materials and Methods

### 2.1. Compliance with Ethics Guidelines

This study was performed in accordance with the principles of the Declaration of Helsinki, and with the Ethical Guidelines for Medical and Health Research Involving Human Subjects. All participants were given a full explanation of the study and informed consent was obtained electronically over the Internet. This study was approved by the Institutional Review Board of TOUKEIKAI Kitamachi Clinic (approval number: STS07778 and date: 16 December 2020) and was registered with UMIN-CTR (http://www.umin.ac.jp/ accessed on 23 December 2021, Identification No. UMIN000042790).

### 2.2. Study Design and Participants

This study was a web-based cross-sectional survey conducted in December 2020. Participants were recruited by an internet research company (Rakuten Insight, Inc., Tokyo, Japan). Inclusion criteria were as follows: (1) 20 years of age or older, (2) diagnosed with DED and prescribed one type of DED eye drops (i.e., one of DQS, HA, or RBM), and (3) eye drop instillation for more than 1 month prior to enrollment. The following individuals were excluded: (1) medical professionals (doctors, pharmacists, nurses, etc.), (2) those engaged in the pharmaceutical or medical device industries, (3) those engaged in pharmaceutical distribution, (4) those engaged in information provision services, research services, or advertising, (5) those who had DED complicated with diabetes or ocular infections, (6) those who had glaucoma or allergic conjunctivitis treated by eye drop instillation, (7) those who had undergone ophthalmic surgery within 1 month prior to providing informed consent, (8) those who had a history of punctual plug or surgical procedure, (9) those who did not visit an ophthalmologist for the purpose of DED treatment within 3 months prior to providing informed consent, or whose timing of their last visit was unknown, and (10) those who instilled two or more types of DED eye drops (DQS, HA, and RBM) concurrently. Participants who met all the inclusion criteria and none of the exclusion criteria were included in the study. The sample size for this study was 1000 participants for each of the three DED eye drops (DQS, HA, and RBM), for a total of 3000 participants.

### 2.3. Data Collection and Management

An e-mail was sent to each participant enrolled in the study, including links to the web site for a one-time web-based questionnaire. Participants filled in the questionnaire as a self-completion survey with instructions provided for each question, and response data were recorded, collected electronically, and stored in a database managed by Rakuten Insight. The collected data were anonymized at Rakuten Insight. Then, an internet research support company (M3 Inc., Tokyo, Japan) edited, cleaned, and reviewed the results, and created a database of the collected data.

### 2.4. Questionnaire

The questionnaire consisted of 19 questions, which were as follows: questions about patient background (Q1–8), questions about the treatment for DED in the last 3 months (Q9–12), a question about the frequency of instillation per day for the treatment of DED in the last 1 month (Q13), a question about the relation between their subjective symptoms and the instillation of the DED eye drops (Q14), questions about DED eye drops usage as instructed by the ophthalmologists or pharmacists (Q15, Q16), if applicable, the reasons why participants did not instill the DED eye drops as recommended by the ophthalmologists or pharmacists (Q17, 18), and the intensity of the subjective symptom(s) of DED before treatment and in the last 1 month (Q19). The content of the questionnaire is shown in File S1.

The following information was collected on patient background (Q1–8): age, sex, daily contact lens use, the duration of video display terminal (VDT) use in daily life, smoking behavior status, years after diagnosis of DED, and presence of ocular or systemic complication. The following information was collected on the treatment for DED (Q9–12): type of DED eye drops, the frequency of instillation per day in the last 1 month, the duration of treatment for DED, treatment for DED other than DQS, HA, or RBM.

Regarding the reasons why participants did not instill the DED eye drops at the frequency at which the ophthalmologists or pharmacists instructed (Q18), participants were presented with 11 reasons and an open-ended box, and asked to rate each reason on a 4-point scale (Strongly agree, Agree, Disagree, Strongly disagree). This question was answered only by the participants who had reported at least one day when they were unable to use the DED eye drops at the frequency stated in the medication instruction.

The intensity of the subjective symptoms (eye fatigue, dryness, discomfort, etc.) was evaluated before using the DED eye drops (before treatment) and during this study (after treatment) on a 5 point scale: 0, No symptoms; 1, Mild; 2, Moderate; 3, Severe; and 4, Very severe. Additionally, the mean scores at each time point and the scores of the amount of change in symptoms from “before treatment” to “after treatment” were calculated.

### 2.5. Comparison of the Fixed-Use Group with the Non-Fixed Use Group

Regular instillation of DED eye drops at the specified frequency is important to break the vicious cycle and to improve DED symptoms. However, the characteristics of the population who regularly instill at the specified frequency are not clear. In order to confirm whether there is a difference in background information and symptoms between those who instill at the specified frequency (the fixed-use group) and those who do not (the non-fixed use group), participants were divided into those who instilled the DED eye drops at the specified frequency regardless of whether they had subjective symptoms (fixed use) and all other participants (non-fixed use). The specified frequency for each DED eye drop was as follows: DQS, 6 times per day; HA, 5 to 6 times per day; RBM, 4 times per day.

### 2.6. Statistical Analysis

All statistical analyses were performed using IBM SPSS Statistics 25.0 (IBM Corp, Armonk, New York, NY, USA). Aspin-Welch’s *t*-tests were performed for serial data, and the Fisher’s exact test or the Wilcoxon’s rank sum test were performed for categorical data. Two-sided *p*-values < 0.05 were considered significant.

In the second analysis to understand the effect of the different usage of DED eye drops on subjective symptom improvement, a multiple regression analysis was performed. Covariates were adjusted to compare the fixed-use group and the non-fixed use group. Covariates were selected based on the results of multiple regression analysis results using a stepwise method to determine factors related to fixed-use outcomes and non-fixed use outcomes. A multiple regression analysis to compare the fixed-use group and the non-fixed use group was then performed using statistically calculated covariates, in addition to sex and VDT hours as clinical factors.

## 3. Results

### 3.1. Characteristics of the Study Participants

In total, 2645 participants were enrolled. The target sample size was reached by the scheduled date for participants who instilled DQS (*n* = 1100), and HA (*n* = 1100), but not for participants who instilled RBM. The extension of recruitment to the end of December resulted in the enrollment of 445 RBM participants. No data were missing for any participant.

Table 1 shows the background characteristics of the participants. The most common age group was 50–59 years (26.4%) followed by 40–49 years (22.2%). There were 1082 males (40.9%) and 1563 females (59.1%). Median duration after diagnosis of DED was 6.1 ± 6.1 years. The most common duration of treatment for DED was at least 3 years (33.8%), followed by 1 year or more and less than 3 years (30.5%).

Tables S1 and S2  show the characteristics of patients, divided into three groups by DED eye drop type. Participants who used HA were older (most commonly 40–69 years old) and tended to have a longer duration of DED treatment. A greater proportion of participants who used RBM tended to have a history of smoking and to use concomitant medications, such as steroid eye drops and over-the-counter (OTC) eye drops.

### 3.2. Frequency of Instillation of DED Eye Drops: Instillation Behavior and Medication Instruction

**Figure 1:**
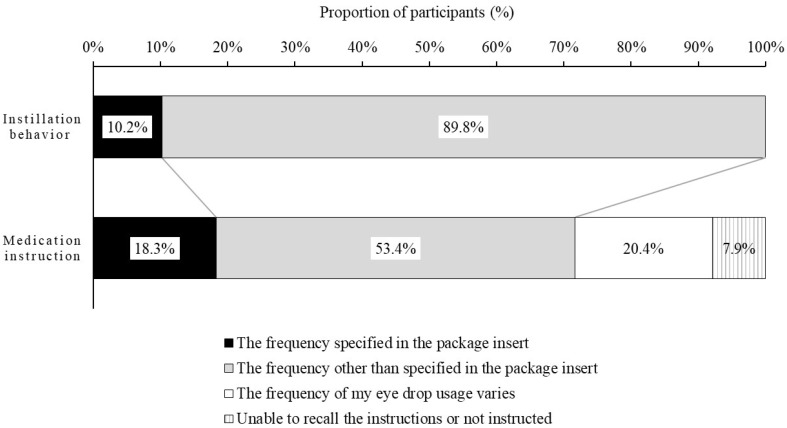
Distribution of participants according to actual frequency of instillation and medication instruction knowledge status. The frequency specified in the package insert for each DED eye drop was as follows: DQS, 6 times per day; HA, 5 to 6 times per day; RBM, 4 times per day. DQS: 3% diquafosol sodium ophthalmic solution; HA: sodium hyaluronate ophthalmic solution; RBM: 2% rebamipide ophthalmic suspension; DED: dry eye disease.

Figure 1 shows the distribution of participants according to the actual frequency of instillation and medication instruction knowledge status. In all, 10.2% of participants instilled the eye drops at the specified frequency and 18.3% of participants knew or were cognizant of the specified frequency instructed by their ophthalmologist or pharmacists.

The distribution of participants according to the actual frequency of instillation for the three types of DED eye drops is shown in Figure S1 and the distribution of participants according to medication instruction knowledge status for the three types of DED eye drops is shown in Table S3. The proportion of participants who actually instilled the DED eye drops at the specified frequency (4 times per day) was high (13.7%) for those instilling RBM and low for those instilling HA and DQS. On the other hand, the proportion of participants who answered “at the specified frequency instructed by their ophthalmologist or pharmacists,” was the highest for RBM (28.8%), but patients in the HA group were less likely to know the specified frequency than patients in the DQS group.

### 3.3. Reasons Why Participants Did Not Instill the Eye Drops at the Frequency Instructed by Ophthalmologists or Pharmacists

**Figure 2:**
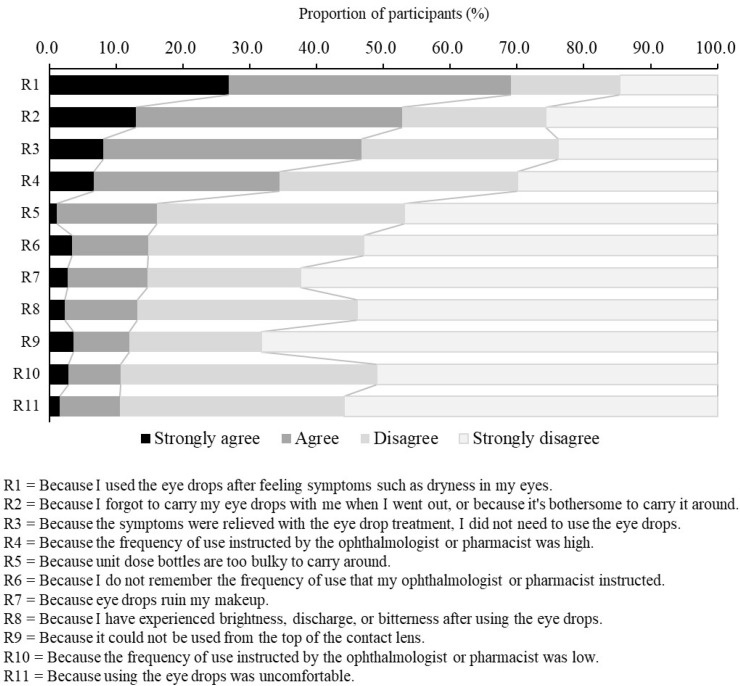
Reasons participants gave for not instilling the eye drops at the frequency instructed by ophthalmologists or pharmacists. The 11 reasons were evaluated on a 4 point scale (Strongly agree, Agree, Disagree, Strongly disagree). DED: dry eye disease.

Figure 2 shows the proportions of participants grouped by their reasons for not following medication instructions. These data were from the 2108 participants (79.7%) who had answered that there was at least one day when DED eye drops were not used at the specified frequency. Participants “agreed” or “strongly agreed” with the following reasons: R1 (Because I used the eye drops after feeling symptoms such as dryness in my eyes.), R2 (Because I forgot to carry my eye drops with me when I went out, or because it’s bothersome to carry it around.), R3 (Because the symptoms were relieved with the eye drop treatment, I did not need to use the eye drops.), R4 (Because the frequency of use instructed by the ophthalmologist or pharmacist was high.), and R5 (Because unit dose bottles are too bulky to carry around.). There was no difference in the proportions of patients by reason for not following medication instructions among the three groups categorized by eye drop type (Figure S2). Among participants who received instruction from ophthalmologists or pharmacists, the reasons given for not following instructions in the order of highest to lowest proportion of participants was R1, R2, R4, R3, and R5. In this population, the frequency of the R4 response was higher (Figure S3).

### 3.4. Usage Pattern of Eye Drops for Subjective Symptoms: Instillation Behavior and Medication Instruction

We considered that it was important to instill eye drops not only at the fixed frequency but also regardless of the symptoms, and so we examined the relationship between eye drop use and subjective symptoms.

**Figure 3:**
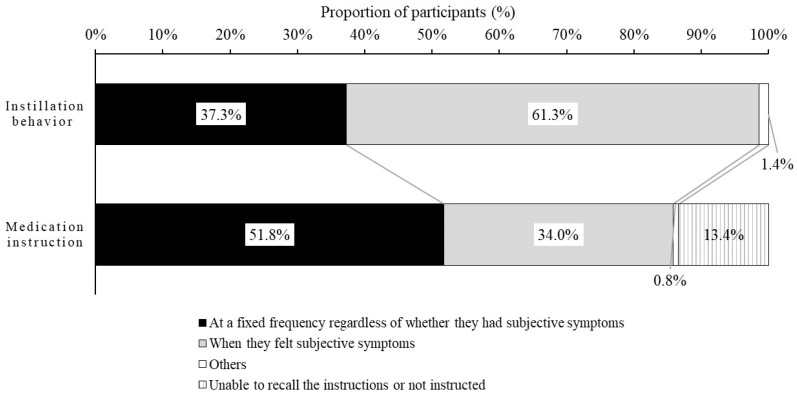
Distribution of participants who instilled according to actual usage pattern and participants who indicated the usage pattern instructed by their ophthalmologist or pharmacists. Subjective symptoms related to DED represented dryness, eye fatigue, etc. DED: dry eye disease.

Figure 3 shows the distribution of participants according to actual usage pattern for subjective symptoms and medication instruction knowledge status. Regarding the actual usage pattern of participants, 61.3% of participants instilled the DED eye drops when they felt subjective symptoms. In contrast, regarding the usage pattern in the medication instruction, 51.8% of participants were instructed to use the DED eye drops at the specified frequency regardless of subjective symptoms.

The distribution of participants according to actual usage pattern and medication instruction knowledge status for the three types of DED eye drops is shown in Table S4. There was no difference in the proportion of patients according to the actual usage pattern and medication instruction knowledge status among the three groups categorized by eye drop type.

### 3.5. Comparison of the Fixed-Use Group with the Non-Fixed Use Group

Regular instillation of the DED eye drops at the specified frequency is important to break the vicious cycle and to improve DED symptoms. However, the characteristics of the population who regularly instill at the specified frequency (the fixed-use group) are not clear. We investigated the difference in background information and symptoms between the fixed-use group and the other group (the non-fixed-use group). There were 153 participants (5.8%) assigned to the fixed-use group and 2492 participants (94.2%) assigned to the non-fixed use group. Table 2 shows the patient background information for the fixed-use group and the non-fixed use group. The participants in the fixed-use group were older (*p* = 0.03), had a lower rate of contact lens use (*p* < 0.01), a lower rate of treatment for DED other than treatment for DED with DQS, HA, or RBM (*p* < 0.01), and a higher frequency (days per month) of eye drop usage as treatment for DED (*p* <0.01) than the non-fixed use group (Table 2).

In the comparison between the fixed-use group and the non-fixed use group for the three eye drop types (DQS, HA, and RBM), there were no between-group differences in patient background factor distribution (Tables S5 and S6).

**Figure 4:**
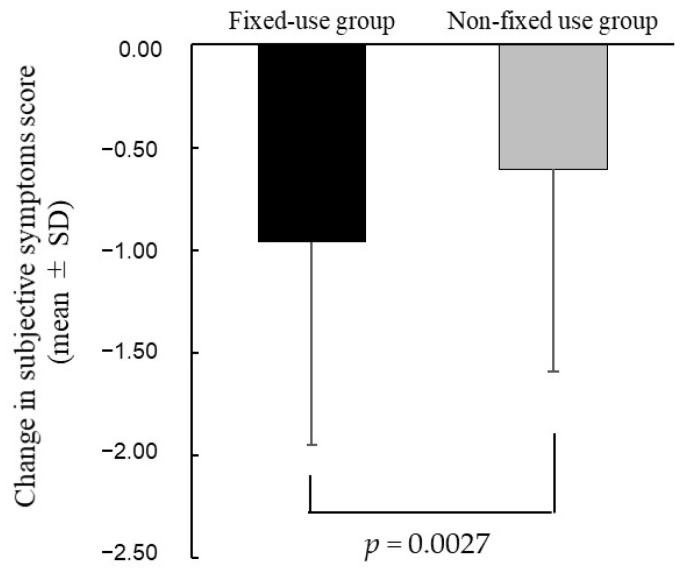
Comparing the change in subjective symptoms score in the fixed-use group with that in the non-fixed use group. Sex, age, VDT hours, use of over-the-counter eye drops, frequency of instillation of DED eye drops (days per month), and the score of the subjective symptoms before treatment were adjusted in the multiple regression model.

Figure 4 compares the subjective symptoms scores of the two groups. The score in the fixed-use group improved from 2.09 ± 1.13 (before treatment) to 1.13 ± 0.72 (after treatment), and the score in the non-fixed-use group improved from 1.81 ± 0.99 to 1.20 ± 0.72. The improvement in subjective symptoms score was significantly greater in the fixed-use group than the non-fixed use group (*p* = 0.0027, Figure 4).

When comparing subjective symptoms scores for each of the three eye drop types (DQS, HA, and RBM) in the fixed-use group and the non-fixed use group, the improvement in the subjective symptoms score was significantly greater in the fixed-use group than the non-fixed use group for DQS and HA (*p* = 0.0302, *p* = 0.0196), but was not different for RBM (*p* = 0.9984, Figure S4).

## 4. Discussion

We showed that most participants with DED did not instill the DED eye drops at the specified frequency, and were not instructed about the specified frequency by ophthalmologists or pharmacists. The most common reason for not instilling the DED eye drops at the frequency specified by medication instruction was the use of eye drops on an as-needed basis only to alleviate subjective symptoms (dryness, eye fatigue, etc.). In addition, more than 60% of participants instilled them when subjective symptoms became apparent. In addition, the improvement in the subjective symptoms score was significantly greater in the fixed-use group than the non-fixed use group, and the non-fixed use group was found to be younger, more likely to wear contact lenses (CLs), and more likely to use the OTC eye drops.

Our study investigated the actual frequency of instillation in patients use and the perception of patients with the specified frequency instructed by their ophthalmologist or pharmacists. The actual frequency of instillation by patients was less than that specified in the package insert, and eye drops were instilled at the specified frequency in only about 10% of patients. Similar to our study, the study of Eguchi et al. evaluated the frequency of eye drop instillation in patients with DED (most patients were 20–30 years old; more than half of patients used artificial tears) using the DryEyeRhythm smartphone application [14]. In that study, more than half of the patients used eye drops one to three times per day for the treatment of DED, and most patients were unable to instill the DED eye drops at the specified frequency (five to six times per a day for artificial tears). Our study showed that the frequency of instillation of eye drops prescribed for DED in our patients, who were commonly 50–59 years old (older than those in the Eguchi study), was below the specified frequency. Those results indicated that most DED patients did not instill the DED eye drops at the specified frequency in Japan.

In addition to the actual instillation behavior, it was surprising that more than 70% to 80% of participants did not know the frequency specified in the package insert. Our results suggest that patients did not instill at the specified frequency because of failure to recognize or adhere to the details of usage provided by ophthalmologists and pharmacists. Another reason is that the ophthalmologists may prescribe a lower frequency of treatment based on the patient’s symptoms and lifestyle. In addition, the trivialization of eye drop usage may lead them to forget the frequency instructed by ophthalmologists and pharmacists. Our results suggest that it is very important to provide patients with information about usage, including the frequency of usage.

The relationship between subjective symptoms and instillation behavior is not clear. Therefore, we investigated the relationship between subjective symptoms and instillation behavior. It was found that 61.3% of participants instilled the DED eye drops to alleviate subjective symptoms. In addition, it was clear that instillation only as needed to alleviate subjective symptoms (e.g., dryness, eye fatigue) was the reason participants did not instill at the frequency instructed by ophthalmologists and pharmacists. If the eye drops are instilled after symptoms develop, the instillation of eye drops may produce temporary symptomatic relief. However, patients may have immediate recurrence of symptoms because the vicious cycle between the abnormal functioning of the corneal epithelium and the instability of tear film layer still exists. This may indicate that it is important to continue using the drug in order to prevent the symptoms from appearing, rather than using the eye drops to suppress the symptoms once they occur. Therefore, it might be desirable for ophthalmologists or pharmacists to ensure, through communication at the clinical site, that patients understand the importance of the continuous use of eye drops, with or without symptoms.

We examined the improvement of subjective symptoms in the patients who regularly instilled at the specified frequency (the fixed-use group). We showed that the improvement in subjective symptoms score was significantly greater in the fixed-use group than the non-fixed use group. Likewise, Asbell et al. compared the clinical effects of fixed versus as-needed administration using artificial tear fluid (polyethylene glycol and propylene glycol; Systane Ultra). Their study showed that the Impact of Dry Eye on Everyday Life scores were significantly improved by fixed use over as-needed use of artificial tears [15]. Thus, it suggested that regular instillation of eye drops at the specified frequency, rather than instillation on an as-needed basis to alleviate subjective symptoms, may affect subjective symptoms. When the improvement in subjective symptoms was compared between the fixed-use group and the non-fixed use group for the three types of DED eye drops (DQS, HA, and RBM), the improvement in subjective symptoms by DQS and HA was significantly greater in the fixed-dose group than in the non-fixed-dose group, but that by RBM was not significantly greater. When comparing steroid eye drop usage among the three groups divided on the basis of DED eye drop type, the frequency of use of steroid eye drops was significantly higher only in the non-fixed use group for RBM. Therefore, differences in concomitant medications may have affected subjective symptoms, and the results for RBM may have differed from the results for DQS and HA.

In addition, we examined the characteristics of the patients who regularly instilled at the specified frequency (the fixed-use group). The participants in the fixed use group were older, had a lower rate of CLs use, and a lower rate of OTC eye drop use. These results suggest that in order to instill regularly at the specified frequency, it may require removal the of factors, such as age, CLs use, and OTC eye drop use, that interfere with instillation behavior, in order to make instillation habitual. On the other hand, as the second reason why participants did not instill at the frequency instructed, a high percentage of participants chose “Because I forgot to carry my eye drops with me when I went out, or because it’s bothersome to carry it around.” Therefore, there are limitations to removing factors that interfere with instillation behavior. Particularly in younger patients, who are busy and have little spare time, and where many factors exist that interfere with instillation behavior, such as the use of CLs and OTC eye drops, as found in this study, it may be necessary for physicians to provide medication instruction emphasizing adherence to the specified frequency of eye drop usage.

In this study, we analyzed the actual use of the DED eye drops and determined the reasons for instillation behavior in terms of medication instruction and subjective symptoms. In addition, subjective symptoms were significantly improved by regular instillation at the specified frequency, and the characteristics of patients in the fixed-use group were confirmed. Understanding the patient’s instillation behavior is extremely important for providing medication instructions. The education of patients through medication instruction may contribute to improving the symptoms and quality of life of DED patients in the future. On the other hand, medication instruction alone for improving adherence to the recommended instillation frequency was found in some cases to be of limited value, because participants thought that the daily frequency of instillation was too high. The frequency of eye drop instillation in Japan is 4–6 times per day, which is too high for most patients [16,17,18]. The specified frequency of eye drop instillation is 1–2 times per day in the United States and Europe [19,20,21]. Therefore, in order to be instilled regularly at the specified frequency, eye drops requiring a lower instillation frequency might be desired in the future for dry eye treatment in Japan.

This study had several limitations, which may be web survey-specific or study-specific. First, web surveys are problematic, because they are limited to Web users, making the results difficult to generalize. Second, objective findings such as tear film break-up time or staining score could not be investigated; therefore, the diagnosis of dry eye was self-reported by the participants. Third, since the responses to questions about the past required participant recall, there was also recall bias. Further studies in patients with a more homogeneous background may be needed to compare the efficacy of fixed use among users of the three types of the DED eye drops. Fourth, the population in this study was a relatively elderly population and included subjects who had continued treatment for at least 10 years. It is unclear whether the reason participants in this study did not instill at the frequency instructed is the same in each age group and for each duration of treatment. In addition, patients who continued treatment for less than one month were not included in this study. The reason for instillation behavior in the newly initiated population is not known to be the same as in this study population. Therefore, further studies may be needed to analyze the actual use of the DED eye drops in DED patients, including studies of associations with age, sex, various subjective symptoms, and objective findings. Moreover, further study of differences between the three DED eye drops (e.g., how differences in the mechanism of action affect different subjective symptoms) may be required in the future.

## 5. Conclusions

Most participants did not instill eye drops at the frequency specified in the package insert, and instead instilled them only as needed to alleviate subjective symptoms. In order to obtain the appropriate effect of eye drops, ophthalmologists need to impress upon patients the importance of regular instillation at the frequency specified in the package insert, while taking patient characteristics such as age and the concomitant medications into account.

## Figures and Tables

**Table 1:** Background characteristics of the participants.

Demographic Characteristics		Total (*n* = 2645)
Age category, *n* (%)		
	20–29	98 (3.7)
	30–39	381 (14.4)
	40–49	586 (22.2)
	50–59	697 (26.4)
	60–69	542 (20.5)
	70–79	323 (12.2)
	80 years and over	18 (0.7)
Male, *n* (%)		1082 (40.9)
Daily CL use, *n* (%)		777 (29.4)
VDT hours, mean ± SD		5.6 ± 3.3
Smoking, *n* (%)		414 (15.7)
Types of DED eye drops, *n* (%)		
	DQS	1100 (41.6)
	HA	1100 (41.6)
	RBM	445 (16.8)
Frequency of instillation of DED eye drops (days per month), n (%)		
	Almost every day	1792 (67.8)
	About 15 days	470 (17.8)
	A few days	277 (10.5)
	Rarely	106 (4.0)
Duration of treatment for DED, *n* (%)		
	1–3 months	289 (10.9)
	3–6 months	330 (12.5)
	6 months to 1 year	325 (12.3)
	1 year to 3 years	806 (30.5)
	At least 3 years	895 (33.8)
Treatment for DED other than DQS, HA, or RBM, *n* (%)		
	Total	1214 (45.9)
	Steroid eye drops	199 (7.5)
	Artificial sodium	281 (10.6)
	Hyaluronic acid (over-the-counter)	422 (16.0)
	Oral drugs	24 (0.9)
	Over-the-counter eye drops	386 (14.6)
	Others	60 (2.3)

SD: standard deviation; DED: dry eye disease; CL: contact lens; VDT: video display terminal; DQS: 3% diquafosol sodium ophthalmic solution; HA: sodium hyaluronate ophthalmic solution; RBM: 2% rebamipide ophthalmic suspension. Oral drugs include any of the following: pregabalin, carbamazepine, duloxetine hydrochloride, and mirogabalin besilate.

**Table 2:** Characteristics in the fixed-use group and the non-fixed use group.

Demographic Characteristics		Fixed-Use Group	Non-Fixed Use Group	*p*-Value
		(*n* = 153)	(*n* = 2492)	
Age category, *n* (%)				
	20–29	1 (0.7)	97 (3.9)	0.03 *
	30–39	20 (13.1)	361 (14.5)	
	40–49	28 (18.3)	558 (22.4)	
	50–59	43 (28.1)	654 (26.2)	
	60–69	39 (25.5)	503 (20.2)	
	70–79	21 (13.7)	302 (12.1)	
	80 years or over	1 (0.7)	17 (0.7)	
Male, *n* (%)		61 (39.9)	1021 (41.0)	0.80
Daily CLs use, *n* (%)		28 (18.3)	749 (30.1)	<0.01 ^#^
VDT hours, mean ± SD		5.7 ± 3.1	5.6 ± 3.3	0.79
Smoking, *n* (%)		17 (11.1)	397 (15.9)	0.14
Years after diagnosis, mean ± SD		5.6 ± 5.6	6.1 ± 6.1	0.44
Type of eye drops as treatment for DED				
	DQS	63 (41.2)	1037 (41.6)	-
	HA	47 (30.7)	1053 (42.3)	
	RBM	43 (28.1)	402 (16.1)	
Frequency of instillation of DED eye drops (days per month), *n* (%)				
	Almost every day	147 (96.1)	1645 (66.0)	<0.01 **
	About 15 days	4 (2.6)	466 (18.7)	
	A few days	1 (0.7)	276 (11.1)	
	Rarely	1 (0.7)	105 (4.2)	
Treatment for DED other than DQS, HA, or RBM, *n* (%)				
	Total	52 (34.0)	1162 (46.6)	<0.01 ^#^
	Steroid eye drops	8 (5.2)	191 (7.7)	0.34
	Artificial sodium	12 (7.8)	269 (10.8)	0.28
	Hyaluronic acid (over-the-counter)	18 (11.8)	404 (16.2)	0.17
	Oral drugs	1 (0.7)	23 (0.9)	1.00
	Over-the-counter eye drops	11 (7.2)	375 (15.0)	<0.01 ^#^

SD: standard deviation; DED: dry eye disease; CLs: contact lenses; VDT: video display terminal. * *p* < 0.05, ** *p* < 0.01, Mann-Whitney U-test. ^#^ *p* < 0.05, Fisher exact test.

## Data Availability

The data presented in this study are available on request from the corresponding author.

## Supplementary Materials

The following supporting information can be downloaded at: https://www.mdpi.com/article/10.3390/jcm11020367/s1, File S1, questionnaire; Table S1, the background information of the participants who used the three types of DED eye drops; Table S2, the treatment details of participants according to DED eye drop type; Table S3, distribution of participants who answered “at the frequency instructed by their ophthalmologist or pharmacists,” for the three types of DED eye drops; Table S4, differences in usage patterns among patients using the three types of DED eye drops; Table S5, the background information of patients in the fixed-use and non-fixed use groups using the 3 DED eye drop types (DQS, HA, and RBM); Table S6, the treatment details of patients in the fixed-use and non-fixed use groups, by eye drop type (DQS, HA, and RBM); Figure S1, distribution of participants according to actual frequency of instillation for the three types of DED eye drops (DQS, HA, and RBM); Figure S2, reasons participants gave for not instilling the eye drops at the frequency instructed by ophthalmologists or pharmacists for the three types of DED eye drops; Figure S3, reasons participants gave for not instilling the eye drops at the frequency instructed by ophthalmologists or pharmacists for the three types of DED eye drops. (Limited to the participants who were instructed to instill at the specified frequency.); Figure S4, change in subjective symptoms score, comparing the fixed-use group with the non-fixed use group using the 3 DED eye drops (DQS, HA, and RBM).
