# Supplementary material for: Adherence to Eye Drops Usage in Dry Eye Patients and Reasons for Non-Compliance: A Web-Based Survey
Source: J Clin Med. 2022 Jan 12;11(2):367. doi: 10.3390/jcm11020367 (PMC8779746; doi:10.3390/jcm11020367)
Supplement: Supplementary file 1 [file jcm-11-00367-s001.zip › Supplementary File/File S1.pdf]

File S1 Questionnaire.

1. Please tell us your age.

1. 19 or younger
2. 20s
3. 30s
4. 40s
5. 50s
6. 60s
7. 70s
8. 80 or over

(Participants who selected No. 1 were excluded.)

2. Please tell us your gender.

1. Male
2. Female

3. Are you currently suffering from any of the systemic diseases listed below? Please choose all items that apply to you.
(Multiple answers are acceptable.)

1. Sjogren's syndrome
2. Diabetes
3. Rheumatoid arthritis
4. Hypothyroidism
5. Hypertension
6. Insomnia
7. Depression illness
8. Connective tissue disease
9. Not applicable

(Participants who selected No. 2 were excluded.)

4. Please choose all of the following ocular diseases for which you are being treated (within the last 3 months).
(Multiple answers are acceptable.)

1. Cataract
2. Glaucoma
3. Dry eye
4. Allergic conjunctivitis
5. Ocular infection
6. Other (answer freely)
7. Not applicable

(Participants who did not select No. 3 and those who selected either No. 5 or No. 7 were excluded.)

5. Do you wear contact lenses in your daily life?

1. Yes
2. No

6. How many hours do you spend using VDT devices in your daily life? (VDT devices refer to personal computers, mobile phones, smartphones, tablets, and game consoles.)
Note) Enter in half-width numeric characters.

1. ______ Hour(s) per day

7. Do you smoke?

1. Yes
2. No

8. How long ago were you diagnosed with dry eye for the first time by an ophthalmologist?

1. ______ Year(s) ____ month(s)

9. (This question was intended for participants who had been treated for dry eye by an ophthalmologist within the last 3 months.)
From the list below, please choose all types of eye drops for dry eye that your ophthalmologist has prescribed and that you are currently using.
Note) Please check your prescription record and your eye drops before answering.

1. 3% diquafosol sodium ophthalmic solution
2. 0.1% or 0.3% sodium hyaluronate ophthalmic solution
3. 2% rebamipide ophthalmic suspension
4. Not applicable
5. I don’t know the name of the eye drops.

(Participants who selected No. 4 or No. 5 were excluded. Participants who selected more than one answer from No. 1, No. 2, and No. 3 were also excluded.)

10. If you visited an ophthalmologist within the last 3 months, how long have you been using the eye drops you are currently using?

1. Less than 1 month
2. 1 month or more
3. 3 months or more
4. 6 months or more
5. 1 year or more
6. 3 years or more

(Participants who selected No. 1 were excluded.)

11. Do you currently take any medications for the treatment of dry eye other than "eye drops for the treatment of dry eye"? Please tell us all that you use to treat your dry eye.
(Multiple answers are acceptable.)

[Steroid eye drops]
Santeson, D.E.X, Visualin, Teikason, Flumetholon, Odomel, Fluorometholone, Rinderon, Sanbetason, betamethasone sodium phosphate, Berbesolone, Linolosal, and Berbesolone F
[Artificial sodium]
Mytear® ophthalmic solution and Soft Santear®
[Hyaluronic acid (Over the counter)]
Hyalein® S
[Oral drugs]
Pregabalin, carbamazepine, duloxetine hydrochloride, mirogabalin besilate
[Over-the-counter eye drops that you are able to purchase yourself, such as at a pharmacy or a drug store]
Eye drops labeled "dry eye" and "tired eye"

1. Steroid eye drops
2. Artificial sodium
3. Hyaluronic acid (over the counter)
4. Oral drugs
5. Over-the-counter eye drops that you are able to purchase yourself, such as at a pharmacy or a drug store
6. Other (answer freely)
7. Not applicable

12. In the last 1 month, how days did you use an eye drop as treatment for dry eye disease?

1. Almost every day
2. About 15 days
3. A few days (1 or 2 days per week).
4. Rarely

13. In the last 1 month, how many times per day did you use an eye drop as treatment for dry eye?

1. 0 times
2. Once
3. Twice
4. 3 times
5. 4 times
6. 5 times
7. 6 times
8. 7 times
9. 8 times or more

14. In the last 1 month, when did you use an eye drop as treatment for dry eye?

1. Instilled the DED eye drops at a fixed frequency regardless of whether I had subjective symptoms
2. Instilled the DED eye drops only when I felt subjective symptoms (dry, tired, etc.)
3. Other (answer freely)

15. Did the ophthalmologist or pharmacist instruct you on how many times a day to use the eye drop of treatment for dry eye?

1. Once
2. Twice
3. 3 times
4. 4 times
5. 5 times
6. 6 times
7. 7 times
8. 8 times or more
9. The frequency of my eye drop usage varies, not a fixed frequency
10. Unable to recall the instructions or was not instructed

16. Did the ophthalmologist or pharmacist instruct you on when you should use the eye drops as treatment for your dry eye?

1. Should use eye drops at a fixed frequency, regardless of whether I had subjective symptoms
2. Should use eye drops only when I felt subjective symptoms (dryness, eye fatigue, etc.)
3. Other (answer freely)
4. Unable to recall the instructions or was not instructed

17. Regarding the eye drops for the treatment of dry eye, how many days were you unable to use the eye drops at the frequency instructed by your ophthalmologist or pharmacist in a month? Enter a number from 0 to 30.

1. ____Days (30 days per month)

18. How strongly do you agree with the following reasons why you did not use the eye drop at the frequency instructed by your ophthalmologist or pharmacist? Please evaluate each reason on a four-point scale (Strongly agree, Agree, Disagree, Strongly disagree).

Note) Question 10 was to be answered only by participants using 2% rebamipide ophthalmic suspension.

The reasons

R1. Because I used the eye drops after feeling symptoms such as dryness in my eyes.
R2. Because I forgot to carry my eye drops with me when I went out, or because it's bothersome to carry it around.
R3. Because the symptoms were relieved with the eye drop treatment, I did not need to use the eye drops.
R4. Because the frequency of use (times per day) instructed by the ophthalmologist or pharmacist was high.
R5. Because unit dose bottles are too bulky to carry around.
R6. Because I do not remember the frequency of use (times per day) that my ophthalmologist or pharmacist instructed.
R7. Because eye drops ruin my makeup.
R8. Because I have experienced brightness, discharge, or bitterness after using the eye drops.
R9. Because it could not be used from the top of the contact lens.
R10. Because the frequency of use (times per day) instructed by the ophthalmologist or pharmacist was low.
R11. Because using the eye drops was uncomfortable.

19. How severe were your eye symptoms (eye fatigue, dryness, and discomfort) before you started to use eye drops as treatment for dry eye disease? Additionally, how severe were your eye symptoms (eye fatigue, dryness, and discomfort) in the last 1 month? Please let us know the average degree of these symptoms in the last 1 month.

a) Before you started to use eye drops as treatment for dry eye disease
b) In the last 1 month

1. No symptoms
2. Mild
3. Moderate
4. Severe
5. Very severe
