# Supplementary material for: Adherence to Eye Drops Usage in Dry Eye Patients and Reasons for Non-Compliance: A Web-Based Survey
Source: J Clin Med. 2022 Jan 12;11(2):367. doi: 10.3390/jcm11020367 (PMC8779746; doi:10.3390/jcm11020367)
Supplement: Supplementary file 1 [file jcm-11-00367-s001.zip › Supplementary File/Table S1. web survey in dry eye patients.pdf]

Table S1. The background information of the participants who used the three types of DED eye drops.

Demographic characteristics	DQS (*n*=1,100)	HA (*n*=1,100)	RBM (*n*=445)
Age category, *n* (%)			
20-29	50 (4.5)	28 (2.5)	20 (4.5)
30-39	177 (16.1)	135 (12.3)	69 (15.5)
40-49	255 (23.2)	233 (21.2)	98 (22.0)
50-59	310 (28.2)	262 (23.8)	125 (28.1)
60-69	207 (18.8)	251 (22.8)	84 (18.9)
70-79	93 (8.5)	182 (16.5)	48 (10.8)
80 years and over	8 (0.7)	9 (0.8)	1 (0.2)
Male, *n* (%)	441 (40.1)	443 (40.3)	198 (44.5)
Daily CL use, *n* (%)	365 (33.2)	279 (25.4)	133 (29.9)
VDT hours, mean ± SD	5.6 ± 3.3	5.6 ± 3.3	5.7 ± 3.3
Smoking, *n* (%)	155 (14.1)	152 (13.8)	107 (24.0)
Years after diagnosis, mean ± SD	5.2 ± 5.4	7.2 ± 6.7	5.6 ± 5.5
Ocular complications, *n* (%)			
Total	120 (10.9)	159 (14.5)	60 (13.5)
Cataract	75 (6.8)	94 (8.5)	36 (8.1)
Glaucoma	5 (0.5)	12 (1.1)	3 (0.7)
Allergic conjunctivitis	17 (1.5)	21 (1.9)	15 (3.4)
Others	28 (2.5)	40 (3.6)	8 (1.8)
Systemic complications, *n* (%)			
Total	295 (26.8)	337 (30.6)	138 (31.0)
Sjögren's syndrome	18 (1.6)	20 (1.8)	8 (1.8)
Rheumatoid arthritis	19 (1.7)	19 (1.7)	6 (1.3)
Hypothyroidism	16 (1.5)	21 (1.9)	9 (2.0)
Hypertension	161 (14.6)	204 (18.5)	71 (16.0)
Insomnia	85 (7.7)	71 (6.5)	52 (11.7)
Depressive illness	50 (4.5)	49 (4.5)	33 (7.4)
Connective tissue disease	20 (1.8)	17 (1.5)	3 (0.7)

SD: standard deviation; DQS: 3% diquafosol sodium ophthalmic solution; HA: sodium hyaluronate ophthalmic solution; RBM: 2% rebamipide ophthalmic suspension; DED: dry eye disease; CL: contact lens; VDT: video display terminal.
