# Supplementary material for: Adherence to Eye Drops Usage in Dry Eye Patients and Reasons for Non-Compliance: A Web-Based Survey
Source: J Clin Med. 2022 Jan 12;11(2):367. doi: 10.3390/jcm11020367 (PMC8779746; doi:10.3390/jcm11020367)
Supplement: Supplementary file 1 [file jcm-11-00367-s001.zip › Supplementary File/Table S2. web survey in dry eye patients.pdf]

Table S2. The treatment details of participants according to DED eye drop type.

**Demographic characteristics, *n* (%)**	**DQS** (*n*=1,100)	**HA** (*n*=1,100)	**RBM** (*n*=445)
Frequency of instillation of DED eye drops (days per month)			
Almost every day	756 (68.7)	759 (69.0)	277 (62.2)
About 15 days	190 (17.3)	190 (17.3)	90 (20.2)
A few days	125 (11.4)	94 (8.5)	58 (13.0)
Rarely	29 (2.6)	57 (5.2)	20 (4.5)
Duration of treatment for DED			
1 - 3 months	133 (12.1)	90 (8.2)	66 (14.8)
3 - 6 months	154 (14.0)	107 (9.7)	69 (15.5)
6 months to 1 year	147 (13.4)	107 (9.7)	71 (16.0)
1 year to 3 years	365 (33.2)	329 (29.9)	112 (25.2)
At least 3 years	301 (27.4)	467 (42.5)	127 (28.5)
Treatment for DED other than DQS, HA, or RBM			
Total	354 (32.2)	652 (59.3)	208 (46.7)
Steroid eye drops	86 (7.8)	52 (4.7)	61 (13.7)
Artificial sodium	106 (9.6)	116 (10.5)	59 (13.3)
Hyaluronic acid (over-the-counter)	-	422 (38.4)	-
Oral drugs	3 (0.3)	8 (0.7)	13 (2.9)
Over-the-counter eye drops	152 (13.8)	135 (12.3)	99 (22.2)
Others	34 (3.1)	21 (1.9)	5 (1.1)

SD: standard deviation; DQS: 3% diquafosol sodium ophthalmic solution; HA: sodium hyaluronate ophthalmic solution; RBM: 2% rebamipide ophthalmic suspension; DED: dry eye disease; Oral drugs: any of the following; pregabalin, carbamazepine, duloxetine hydrochloride, and mirogabalin besilate.
