# Supplementary material for: Adherence to Eye Drops Usage in Dry Eye Patients and Reasons for Non-Compliance: A Web-Based Survey
Source: J Clin Med. 2022 Jan 12;11(2):367. doi: 10.3390/jcm11020367 (PMC8779746; doi:10.3390/jcm11020367)
Supplement: Supplementary file 1 [file jcm-11-00367-s001.zip › Supplementary File/Table S3. web survey in dry eye patients.pdf]

Table S3. Distribution of participants who answered the frequency instructed by their ophthalmologist or pharmacists for the three types of DED eye drops .

**Frequency (times per day) instructed by their ophthalmologist or pharmacists, *n* (%)**	**DQS** (*n*=1,100)	**HA** (*n*=1,100)	**RBM** (*n*=445)
Once	23 (2.1)	13 (1.2)	25 (5.6)
Twice	50 (4.5)	57 (5.2)	66 (14.8)
3 times	199 (18.1)	244 (22.2)	99 (22.2)
4 times	242 (22.0)	279 (25.4)	128 (28.8)
5 times	84 (7.6)	62 (5.6)	6 (1.3)
6 times	219 (19.9)	75 (6.8)	4 (0.9)
7 times	4 (0.4)	3 (0.3)	1 (0.2)
8 times and more	9 (0.8)	3 (0.3)	2 (0.4)
The frequency of my eye drop usage varies, not a fixed frequency	195 (17.7)	269 (24.5)	76 (17.1)
Unable to recall the instructions or not instructed	75 (6.8)	95 (8.6)	38 (8.5)

DQS: 3% diquafosol sodium ophthalmic solution; HA: sodium hyaluronate ophthalmic solution; RBM: 2% rebamipide ophthalmic suspension.
