# Supplementary material for: Adherence to Eye Drops Usage in Dry Eye Patients and Reasons for Non-Compliance: A Web-Based Survey
Source: J Clin Med. 2022 Jan 12;11(2):367. doi: 10.3390/jcm11020367 (PMC8779746; doi:10.3390/jcm11020367)
Supplement: Supplementary file 1 [file jcm-11-00367-s001.zip › Supplementary File/Table S4. web survey in dry eye patients.pdf]

Table S4. Differences in usage patterns among patients using the three types of DED eye drops.

Usage pattern, *n* (%)	DQS (*n*=1,100)	HA (*n*=1,100)	RBM (*n*=445)
Instillation Behavior			
Participants who instilled the DED eye drops at a fixed frequency regardless of whether they had subjective symptoms	431 (39.2)	370 (33.6)	185 (41.6)
Participants who instilled the DED eye drops when they felt subjective symptoms	650 (59.1)	718 (65.3)	254 (57.1)
Others	19 (1.7)	12 (1.1)	6 (1.3)
Medication instruction			
Participants who were instructed to instill the DED eye drops at a fixed frequency regardless of whether they had subjective symptoms	615 (55.9)	513 (46.6)	241 (54.2)
Participants who were instructed to instill the DED eye drops when they felt subjective symptoms	341 (31.0)	395 (35.9)	164 (36.9)
Others	9 (0.8)	10 (0.9)	2 (0.4)
Unable to recall the instructions or were not instructed	135 (12.3)	182 (16.5)	38 (8.5)

Subjective symptoms related to DED represented dryness, eye fatigue, etc. DQS: 3% diquafosol sodium ophthalmic solution; HA: sodium hyaluronate ophthalmic solution; RBM: 2% rebamipide ophthalmic suspension, DED: dry eye disease.
