# Supplementary material for: Adherence to Eye Drops Usage in Dry Eye Patients and Reasons for Non-Compliance: A Web-Based Survey
Source: J Clin Med. 2022 Jan 12;11(2):367. doi: 10.3390/jcm11020367 (PMC8779746; doi:10.3390/jcm11020367)
Supplement: Supplementary file 1 [file jcm-11-00367-s001.zip › Supplementary File/Table S5. web survey in dry eye patients.pdf]

**Table S5. The background information of patients in the fixed-use and non-fixed use groups using the 3 DED eye drop types (DQS, HA, and RBM).**

| Demographic Characteristics | DQS | | | HA | | | RBM | | |
|---|---|---|---|---|---|---|---|---|---|
| | Fixed-Use Group | Non-Fixed Use Group | *p*-Value | Fixed-Use Group | Non-Fixed Use Group | *p*-Value | Fixed-Use Group | Non-Fixed Use Group | *p*-Value |
| Number of participants analyzed | 63(5.7) | 1037(94.2) | | 47(4.3) | 1053(95.7) | | 43(9.7) | 402(90.3) | |
| Age category, *n* (%) | | | | | | | | | |
| 20-29 | 0 (0.0) | 50 (4.8) | 0.42 | 0 (0.0) | 28 (2.7) | 0.38 | 1 (2.3) | 19 (4.7) | < 0.01** |
| 30-39 | 9 (14.3) | 168 (16.2) | | 6 (12.8) | 129 (12.3) | | 5 (11.6) | 64 (15.9) | |
| 40-49 | 17 (27.0) | 238 (23.0) | | 8 (17.0) | 225 (21.4) | | 3 (7.0) | 95 (23.6) | |
| 50-59 | 19 (30.2) | 291 (28.1) | | 11 (23.4) | 251 (23.8) | | 13 (30.2) | 112 (27.9) | |
| 60-69 | 11 (17.5) | 196 (18.9) | | 14 (29.8) | 237 (22.5) | | 14 (32.6) | 70 (17.4) | |
| 70-79 | 7 (11.1) | 86 (8.3) | | 7 (14.9) | 175 (16.6) | | 7 (16.3) | 41 (10.2) | |
| 80 over | 0 (0.0) | 8 (0.8) | | 1 (2.1) | 9 (0.8) | | 0 (0.0) | 1 (0.2) | |
| Male, *n* (%) | 25 (39.7) | 416 (40.1) | 1.00 | 21 (44.7) | 422 (40.1) | 0.55 | 15 (34.9) | 183 (45.5) | 0.20 |
| Daily CL use, *n* (%) | 15 (23.8) | 350 (33.8) | 0.13 | 7 (14.9) | 272 (25.8) | 0.12 | 6 (14.0) | 127 (31.6) | 0.01# |
| VDT hours, mean ± SD | 5.8±3.3 | 5.6±3.3 | 0.66 | 5.8±2.8 | 5.6±3.3 | 0.68 | 5.5±2.9 | 5.7±3.3 | 0.56 |
| Smoking, *n* (%) | 8 (12.7) | 147 (14.2) | 0.85 | 5 (10.6) | 147 (14.0) | 0.67 | 4 (9.3) | 103 (25.6) | 0.01# |
| Years after diagnosis, mean ± SD | 4.8±6.2 | 5.2±5.4 | 0.19 | 6.5±4.9 | 7.2±6.8 | 0.86 | 5.8±5.3 | 5.6±5.5 | 0.67 |
| Ocular complications, *n* (%) | | | | | | | | | |
| Total | 6 (9.5) | 114 (11.0) | 0.84 | 5 (10.6) | 154 (14.6) | 0.53 | 9 (20.9) | 51 (12.7) | 0.16 |
| Cataract | 4 (6.3) | 71 (6.8) | 1.00 | 5 (10.6) | 89 (8.5) | 0.59 | 5 (11.6) | 31 (7.7) | 0.37 |
| Glaucoma | 0 (0.0) | 5 (0.5) | 1.00 | 1 (2.1) | 11 (1.0) | 0.41 | 0 (0.0) | 3 (0.7) | 1.00 |
| Allergic conjunctivitis | 0 (0.0) | 17 (1.6) | 0.62 | 0 (0.0) | 21 (2.0) | 1.00 | 1 (2.3) | 14 (3.5) | 1.00 |
| Others | 2 (3.2) | 26 (2.5) | 0.67 | 0 (0.0) | 40 (3.8) | 0.41 | 4 (9.3) | 4 (1.0) | < 0.01# |
| Systemic complication, *n* (%) | | | | | | | | | |
| Total | 21 (33.3) | 274 (26.4) | 0.24 | 13 (27.7) | 324 (30.8) | 0.75 | 13 (30.2) | 125 (31.1) | 1.00 |
| Sjögren's syndrome | 3 (4.8) | 15 (1.4) | 0.08 | 1 (2.1) | 19 (1.8) | 0.59 | 1 (2.3) | 7 (1.7) | 0.56 |
| Rheumatoid arthritis | 4 (6.3) | 15 (1.4) | 0.02# | 0 (0.0) | 19 (1.8) | 1.00 | 3 (7.0) | 3 (0.7) | 0.01 |
| Hypothyroidism | 1 (1.6) | 15 (1.4) | 0.61 | 1 (2.1) | 20 (1.9) | 0.60 | 1 (2.3) | 8 (2.0) | 0.60 |
| Hypertension | 11 (17.5) | 150 (14.5) | 0.47 | 11 (23.4) | 193 (18.3) | 0.44 | 8 (18.6) | 63 (15.7) | 0.66 |
| Insomnia | 5 (7.9) | 80 (7.7) | 0.81 | 1 (2.1) | 70 (6.6) | 0.36 | 1 (2.3) | 51 (12.7) | 0.04# |
| Depressive illness | 3 (4.8) | 47 (4.5) | 0.76 | 0 (0.0) | 49 (4.7) | 0.26 | 2 (4.7) | 31 (7.7) | 0.76 |
| Connective tissue disease | 3 (4.8) | 17 (1.6) | 0.10 | 1 (2.1) | 16 (1.5) | 0.53 | 0 (0.0) | 3 (0.7) | 1.00 |

CL: contact lens; SD: standard deviation; DQS: 3% diquafosol sodium ophthalmic solution; HA: sodium hyaluronate ophthalmic solution; RBM: 2% rebamipide ophthalmic suspension; DED: dry eye disease; VDT: video display terminal. *P<0.05, **P<0.01, Mann-Whitney U-test. #P<0.05, Fisher exact test.
