# Supplementary material for: Adherence to Eye Drops Usage in Dry Eye Patients and Reasons for Non-Compliance: A Web-Based Survey
Source: J Clin Med. 2022 Jan 12;11(2):367. doi: 10.3390/jcm11020367 (PMC8779746; doi:10.3390/jcm11020367)
Supplement: Supplementary file 1 [file jcm-11-00367-s001.zip › Supplementary File/Table S6. web survey in dry eye patients.pdf]

Table S6. The treatment details for patients in the fixed-use and non-fixed use groups by eye drop type (DQS, HA, and RBM).

Background factors	DQS			HA			RBM		
	Fixed-Use Group (*n*=63)	Non-Fixed Use Group (*n*=1,037)	*p*-Value	Fixed-Use Group (*n*=47)	Non-Fixed Use Group (*n*=1,035)	*p*-Value	Fixed-Use Group (*n*=43)	Non-Fixed Use Group (*n*=402)	*p*-Value
Frequency of instillation of DED eye drops (days per month), *n* (%)									
Almost every day	61 (96.8)	695 (67.0)	<0.01**	44 (93.6)	715 (67.9)	< 0.01**	41 (97.7)	235 (58.5)	<0.01**
About 15 days	1 (1.6)	189 (18.2)		2 (4.3)	188 (17.9)		1 (2.3)	89 (22.1)	
A few days	1 (1.6)	124 (12.0)		0 (0.0)	94 (8.9)		0 (0.0)	58 (14.4)	
Rarely	0 (0.0)	29 (2.8)		1 (2.1)	56 (5.3)		0 (0.0)	20 (5.0)	
Duration of treatment for DED, *n* (%)									
1 - 3 months	7 (11.1)	126 (12.2)	0.72	3 (6.4)	87 (8.3)	0.18	9 (20.9)	57 (14.2)	0.28
3 - 6 months	11 (17.5)	143 (13.8)		2 (4.3)	105 (10.0)		4 (9.3)	65 (16.2)	
6 months to 1 year	8 (12.7)	139 (13.4)		5 (10.6)	102 (9.7)		2 (4.7)	69 (17.2)	
1 year to 3 years	21 (33.3)	344 (33.2)		13 (27.7)	316 (30.0)		11 (25.6)	101 (25.1)	
At least 3 years	16 (25.4)	285 (27.5)		24 (51.1)	443 (42.1)		17 (39.5)	110 (27.4)	
Treatment for DED other than DQS, HA, or RBM, *n* (%)									
Total	10 (15.9)	274 (26.4)	< 0.01##	30 (63.8)	622 (59.1)	0.55	12 (27.9)	196 (48.8)	< 0.01##
Steroid eye drops	5 (7.9)	81 (7.8)	0.02#	2 (4.3)	50 (4.7)	1.00	1 (2.3)	7 (1.7)	0.02#
Artificial sodium	1 (1.6)	105 (10.1)	1.00	7 (14.9)	109 (10.4)	0.33	4 (9.3)	3 (0.7)	0.63
Hyaluronic acid (over-the-counter)	-	-	-	18 (38.3)	404 (38.4)	1.00	-	-	-
Oral drugs	0 (0.0)	3 (0.3)	0.04#	0 (0.0)	8 (0.8)	0.02#	1 (2.3)	12 (3.0)	1.00
Over-the-counter eye drops	3 (4.8)	149 (14.4)	0.72	1 (2.1)	134 (12.7)	0.05	7 (16.3)	92 (22.9)	0.44
Others	1 (1.6)	33 (3.2)	< 0.01##	3 (6.4)	18 (1.7)	0.55	0 (0.0)	3 (0.7)	1.00

DQS: 3% diquafosol sodium ophthalmic solution; HA: sodium hyaluronate ophthalmic solution; RBM: 2% rebamipide ophthalmic suspension; DED: dry eye disease; Oral drugs: pregabalin, carbamazepine, duloxetine hydrochloride, mirogabalin besilate. **P<0.01, Mann-Whitney U-test. #P<0.05, ##P<0.01, Fisher exact test.
