# Supplementary figures and images for: Adherence to Eye Drops Usage in Dry Eye Patients and Reasons for Non-Compliance: A Web-Based Survey
Source: J Clin Med. 2022 Jan 12;11(2):367. doi: 10.3390/jcm11020367 (PMC8779746; doi:10.3390/jcm11020367)

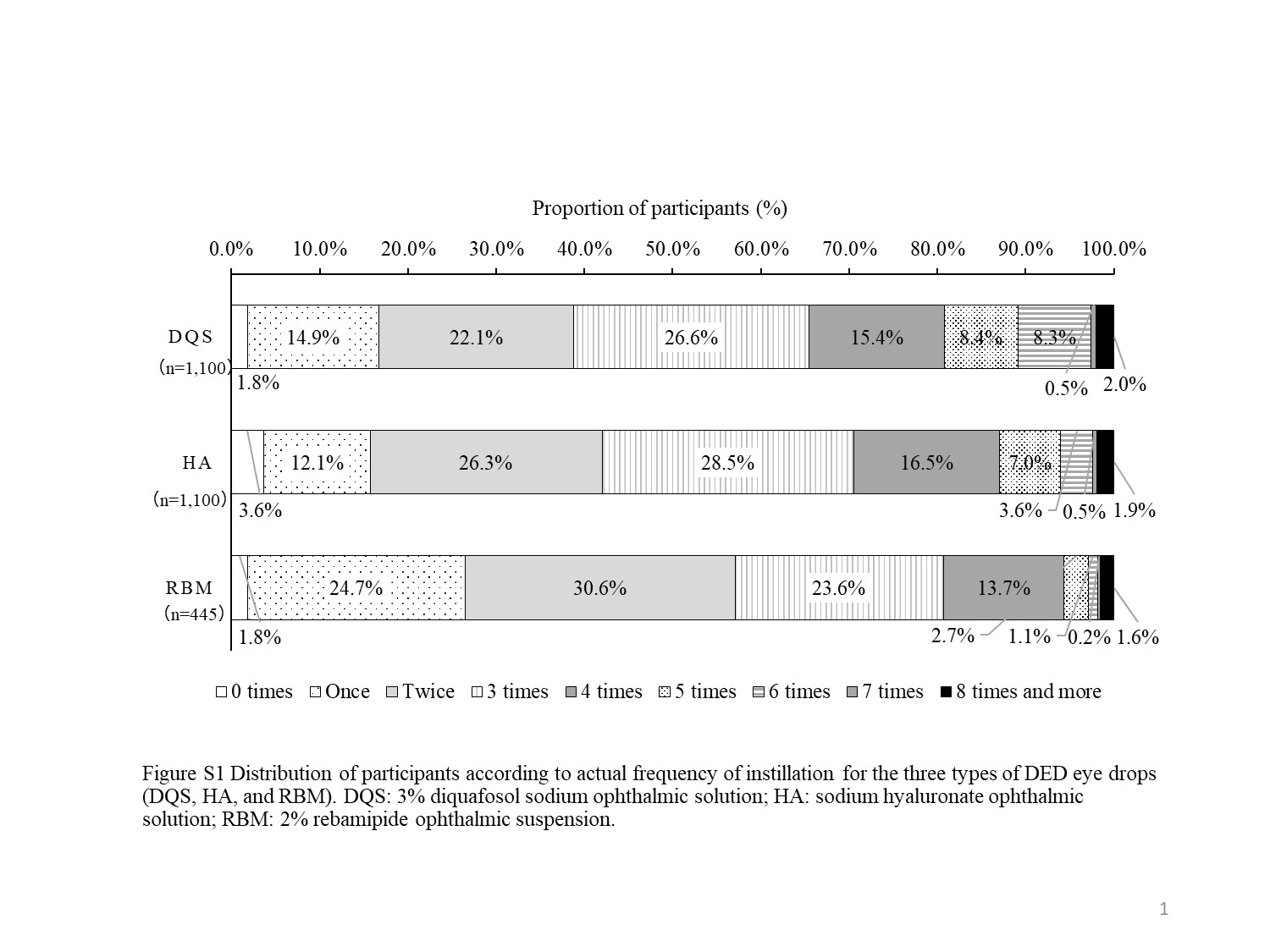

Supplement: Supplementary file 1 [file jcm-11-00367-s001.zip › Supplementary File/Figure S1. web survey in dry eye patients.jpg]

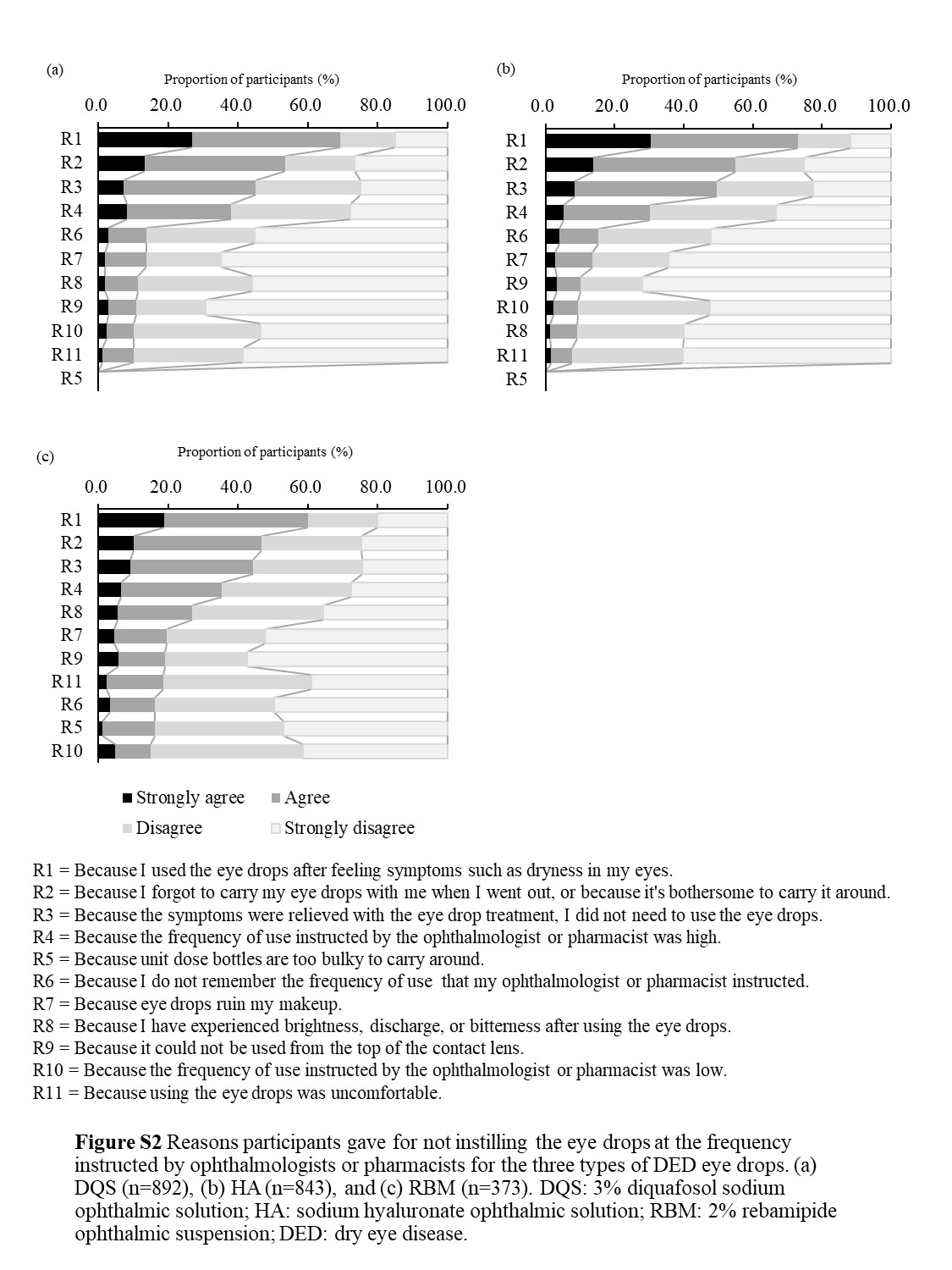

Supplement: Supplementary file 1 [file jcm-11-00367-s001.zip › Supplementary File/Figure S2. web survey in dry eye patients.JPG]

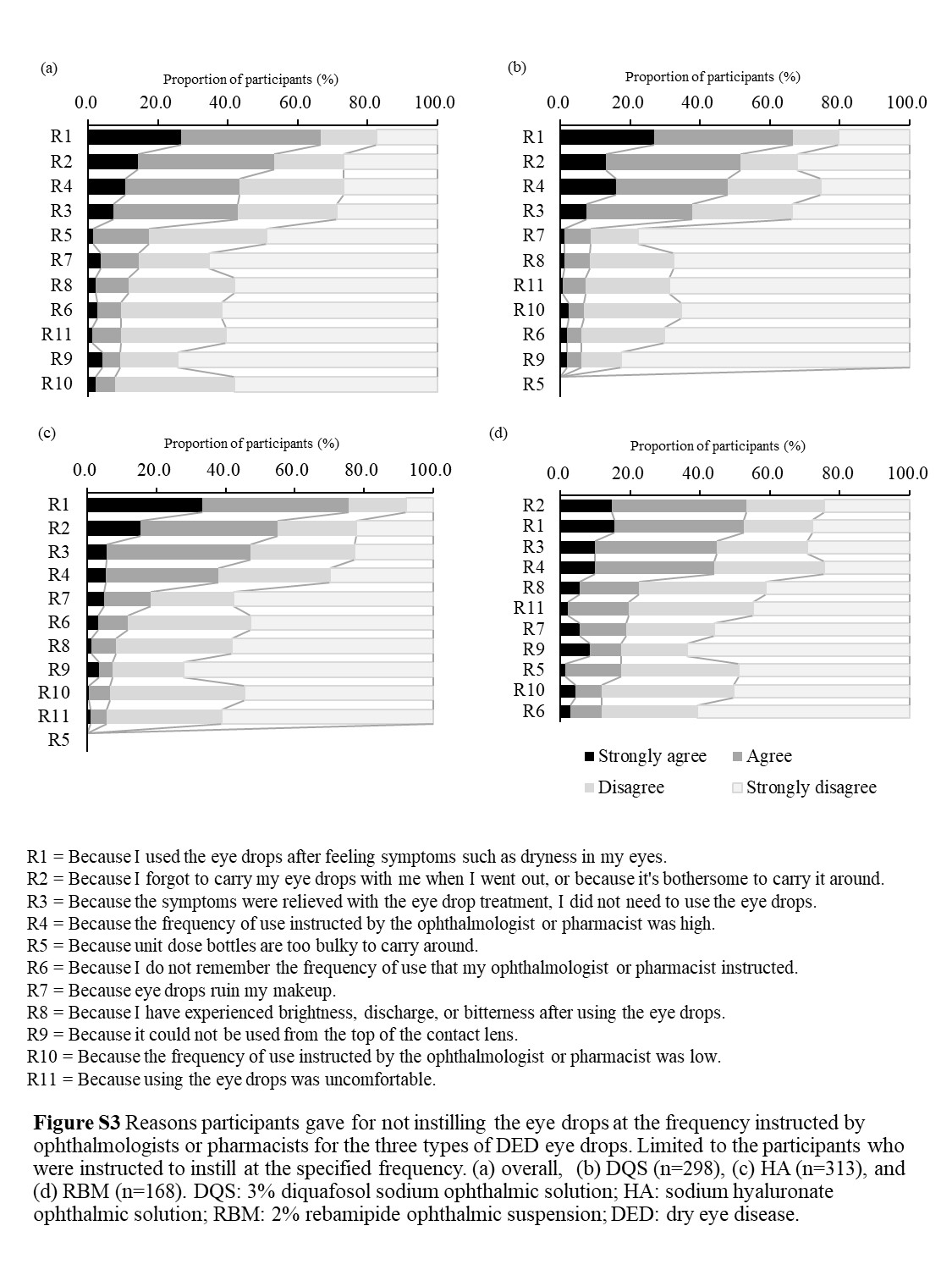

Supplement: Supplementary file 1 [file jcm-11-00367-s001.zip › Supplementary File/Figure S3. web survey in dry eye patients.jpg]

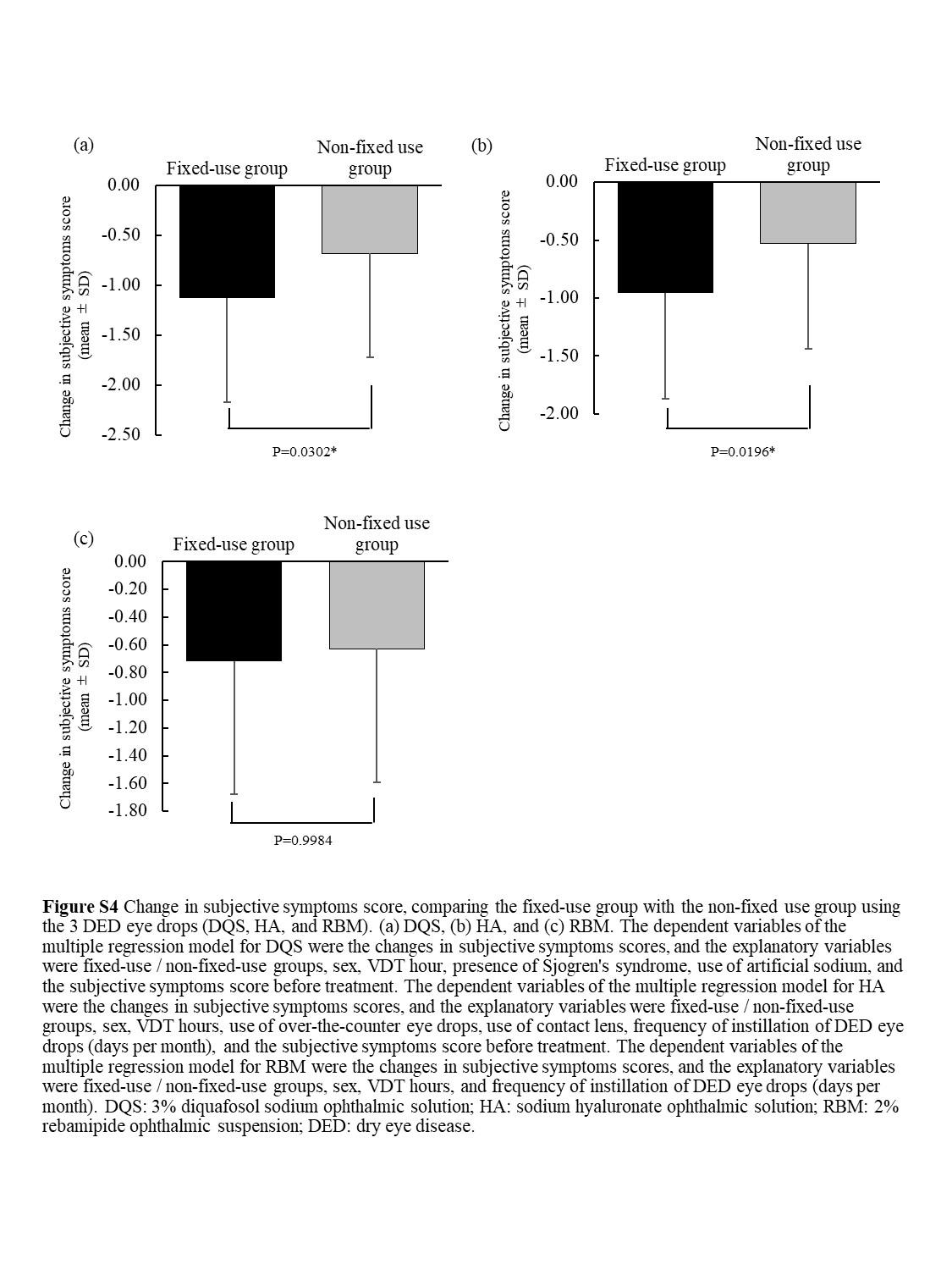

Supplement: Supplementary file 1 [file jcm-11-00367-s001.zip › Supplementary File/Figure S4. web survey in dry eye patients.JPG]
